# The Effect of Vector Silencing during Picornavirus Vaccination against Experimental Melanoma and Glioma

**DOI:** 10.1371/journal.pone.0162064

**Published:** 2016-08-25

**Authors:** Courtney S. Malo, Danielle N. Renner, April M. Huseby Kelcher, Fang Jin, Michael J. Hansen, Kevin D. Pavelko, Aaron J. Johnson

**Affiliations:** 1 Department of Immunology, Mayo Clinic, Rochester, MN, United States of America; 2 Mayo Graduate School, Mayo Clinic, Rochester, MN, United States of America; 3 Neurobiology of Disease Graduate Program, Mayo Clinic, Rochester, MN, United States of America; 4 Department of Neurology, Mayo Clinic, Rochester, MN, United States of America; University of Pécs Medical School, HUNGARY

## Abstract

Virus vector-based vaccination against tumor-specific antigens remains a promising therapeutic approach to overcome the immune suppressive tumor microenvironment. However, the extent that the desired CD8 T cell response against the targeted tumor antigen is impacted by the CD8 T cell response against the virus vector is unclear. To address this question, we used picornavirus vaccination with Theiler’s murine encephalomyelitis virus (TMEV) as our vector against tumor-expressed ovalbumin (OVA_257-264_) antigen in both the B16-OVA murine melanoma and GL261-quad cassette murine glioma models. Prior to vaccination, we employed vector silencing to inhibit the CD8 T cell response against the immunodominant TMEV antigen, VP2_121-130_. We then monitored the resulting effect on the CD8 T cell response against the targeted tumor-specific antigen, ovalbumin. We demonstrate that employing vector silencing in the context of B16-OVA melanoma does not reduce tumor burden or improve survival, while TMEV-OVA vaccination without vector silencing controls tumor burden. Meanwhile, employing vector silencing during picornavirus vaccination against the GL261-quad cassette glioma resulted in a lower frequency of tumor antigen-specific CD8 T cells. The results of this study are relevant to antigen-specific immunotherapy, in that the virus vector-specific CD8 T cell response is not competing with tumor antigen-specific CD8 T cells. Furthermore, vector silencing may have the adverse consequence of reducing the tumor antigen-specific CD8 T cell response, as demonstrated by our findings in the GL261-quad cassette model.

## Introduction

Optimizing vaccines to effectively treat tumors remains a critical goal for immunotherapy. Virus vector-based immunotherapies are an attractive option, as viruses have been employed as oncolytics and can be engineered to express patient-specific tumor antigens [1]. The utility of such vaccines has been demonstrated in mouse models using several viral platforms [2,3,4,5,6]. In particular, murine tumor models treated with viruses engineered to enhance CD8 T cell responses against major histocompatibility complex (MHC) class I-restricted, tumor-specific epitopes have resulted in reduced tumor burden [3,5]. These early studies demonstrate that virus vector-based cancer vaccines have promise for therapeutic benefit.

When employing recombinant virus vaccination against tumor specific antigens, two CD8 T cell responses are generated. One response is directed against the targeted tumor antigen of interest. The other response is directed against epitopes derived from the virus vector [3,7,8]. Importantly, the impact of the CD8 T cell response against the virus vector on the response to the encoded tumor antigen is not fully understood [9,10]. It is possible that the virus vector-specific response competes with the response directed toward the targeted, recombinantly-expressed antigen [11]. Competition among CD8 T cells could occur due to multiple factors, including proximity to antigen presenting cells, peptide:MHC class I abundance, or cytokine availability [11,12]. If competition exists, this poses a challenge to the utility of viral vaccines, as the preferred tumor antigen-specific response may be attenuated. Alternatively, it is possible that virus vector specific CD8 T cells may provide an enhancing effect and boost the CD8 T cell response to the targeted antigen. Therefore, if the virus-specific CD8 T cell response is productive, viral vaccines could afford advantages over other immunotherapy strategies.

In order to determine the effect of CD8 T cell responses directed against a picornavirus vector on the targeted tumor-specific antigen, we developed a method to inhibit CD8 T cell responses. Specifically, we silenced the response to the immunodominant VP2_121-130_ peptide antigen of the picornavirus, Theiler’s murine encephalomyelitis virus (TMEV)_,_ the vector utilized for our vaccine platform [3,5]. Our group has shown that the CD8 T cell response against the D^b^:VP2_121-130_ epitope can be silenced by administering VP2_121-130_ peptide intravenously (i.v.) one day prior to infecting with TMEV [13]. Through additional weekly administration of VP2_121-130_ peptide, the D^b^:VP2_121-130_ epitope-specific response can be inhibited indefinitely [13]. Vector silencing therefore enables us to define the effect of inhibiting virus vector-specific CD8 T cell responses *in vivo*. Using vector silencing, we sought to determine if the CD8 T cell response against the picornavirus vector alters the CD8 T cell response to the tumor-specific antigen during vaccination.

In this study, we employ a picornavirus vaccination strategy using TMEV engineered to express ovalbumin antigen, SIINFEKL (OVA_257-264_). We have demonstrated previously that vaccination with TMEV XhoI-OVA8 (TMEV-OVA) reduces tumor burden and improves survival in animals bearing B16-OVA melanoma and GL261-quad cassette glioma tumors, both of which express OVA_257-264_ as a model tumor antigen [3,5]. We have previously demonstrated that control of tumor burden is mediated by the generation of a productive tumor-specific CD8 T cell response [2,5,14]. In this study, we employ vector silencing to define the impact of virus vector-specific CD8 T cell responses in both peripheral and CNS tumor models through quantification of tumor antigen-specific CD8 T cells and control of tumor burden.

## Results

### Vector silencing does not impact the CD8 T cell response against the recombinantly engineered epitope during peripheral picornavirus infection

**Fig 1:**
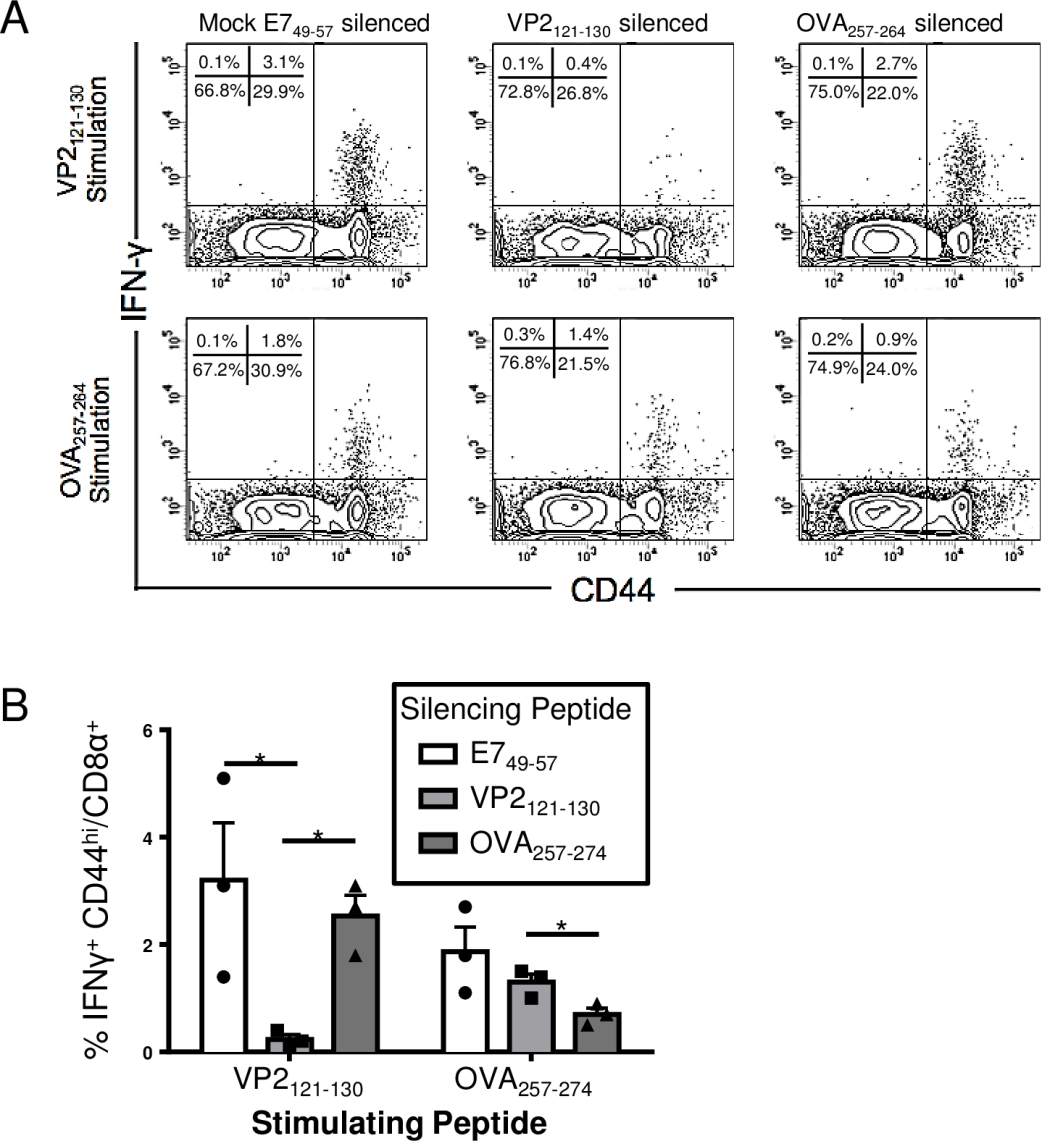
CD8 T cell responses directed against specific epitopes can be silenced during peripheral TMEV-OVA infection. C57BL/6 mice were treated with control E7_49-57_ peptide or silencing VP2_121-130_ peptide. The following day, animals received an i.p. infection of TMEV-OVA. Splenocytes (N = 3 mice per group) were harvested seven days post infection and stimulated with peptide for five hours before intracellular IFNγ staining. (A) Representative flow cytometry plots and (B) average percent of CD8 T cells stimulated to secrete IFNγ are shown. Cells were gated as Viability Dye^lo^, CD45^hi^, and CD8α^+^. Treatment with VP2_121-130_ peptide significantly reduced the VP2_121-130_-specific CD8 T cell responses. Data are shown as mean±SEM. * denotes p<0.05.

Administration of VP2_121-130_ peptide prior to TMEV-OVA infection inhibits the CD8 T cell response against the immunodominant D^b^:VP2_121-130_ epitope in a process we term vector silencing [15]. To determine the extent CD8 T cells specific for the virus vector affect the CD8 T cell response to a recombinantly expressed antigen, we silenced D^b^:VP2_121-130_-specific CD8 T cell responses prior to a peripheral TMEV-OVA challenge [13]. Additionally, we silenced the K^b^:OVA_257-264_-specific CD8 T cell response as a control. We quantified the magnitude of D^b^:VP2_121-130_ and K^b^:OVA_257-264_ epitope-specific CD8 T cell responses seven days post infection (Fig 1). We determined that CD8 T cells isolated from mice silenced with VP2_121-130_ peptide were inhibited from recognizing the D^b^:VP2_121-130_ epitope (Fig 1). However, VP2_121-130_-specific silencing did not result in a change in K^b^:OVA_257-264_-specific CD8 T cells (Fig 1). Likewise, K^b^:OVA_257-264_-specific silencing did not impact the D^b^:VP2_121-130_-specific CD8 T cell response. Therefore, vector silencing does not enhance the response to the recombinantly-expressed epitope seven days post i.p. infection with TMEV-OVA.

### Vector silencing enhances the CD8 T cell response against the targeted OVA_257-264_ antigen of TMEV-OVA during acute CNS infection

**Fig 2:**
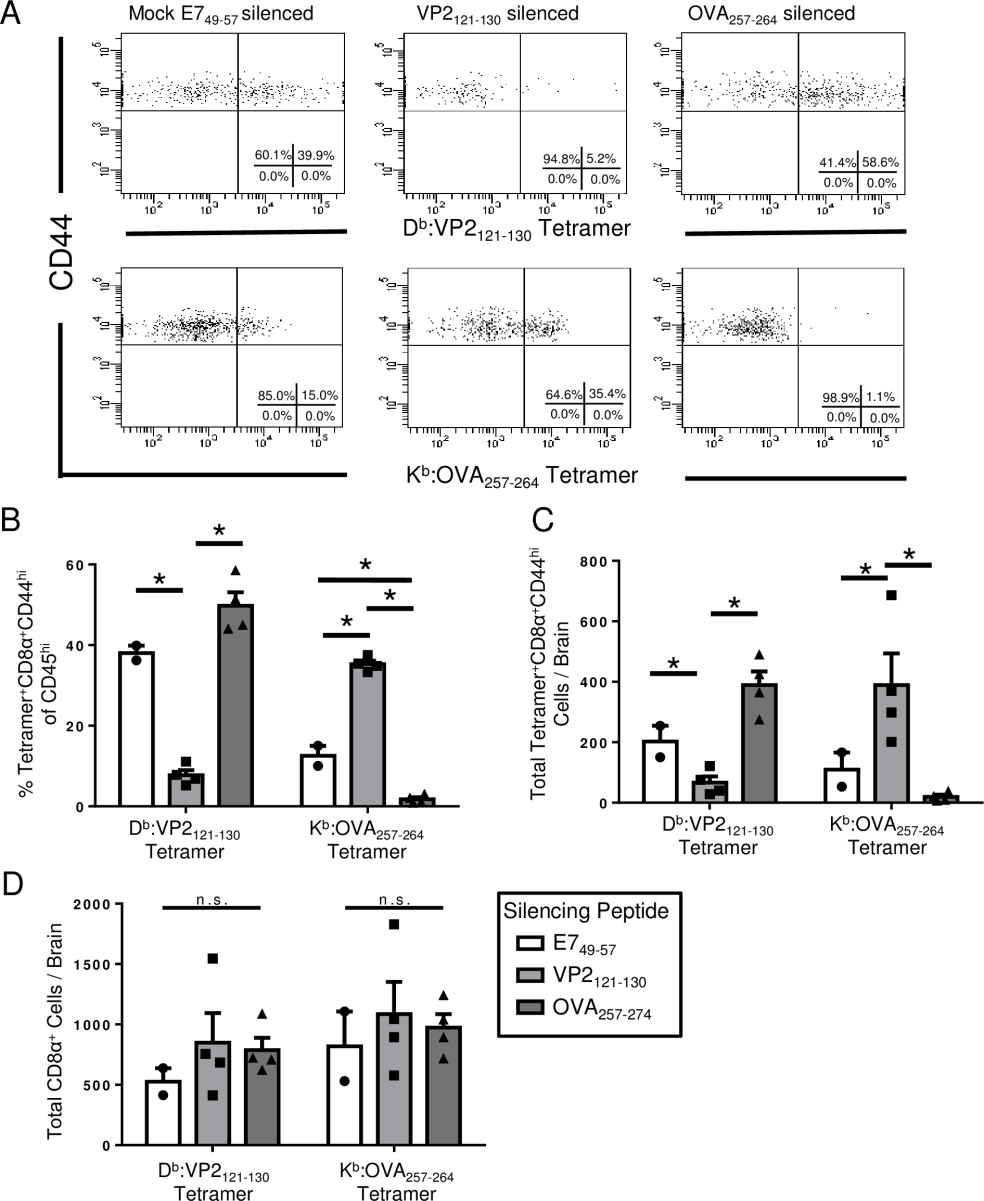
Competition among epitope-specific CD8 T cells occurs in the central nervous system following acute infection with TMEV-OVA. Mice were administered E7_49-57_ (n = 2) or VP2_121-130_ (n = 4) peptide i.v. one day prior to i.c. injection of TMEV-OVA. After one week, brain-infiltrating lymphocytes were harvested and stained for surface markers and peptide:MHC tetramer. (A) Representative flow cytometry plots and (B, C) quantified peptide:MHC tetramer^+^ cells of CD8α^+^CD45^hi^ cells per brain demonstrate a significant decrease in CD8 T cells recognizing VP2_121-130_ upon vector silencing. An increase in peptide:MHC tetramer staining for OVA_257-264_ is also observed. (D) Number of total CD8 T cells infiltrating the brain was not significantly different. Data are shown as mean±SEM. * denotes p<0.05, n.s. indicates no significant difference.

The brain has historically been considered an immune-privileged organ [16,17]. However, it is now accepted that virus infections of the CNS generate robust expansion of CD8 T cells [18,19,20,21,22]. Assessment of picornavirus vaccination in the brain has demonstrated brain-infiltrating CD8 T cell responses against the immunodominant D^b^:VP2_121-130_ epitope and recombinantly expressed K^b^:OVA_257-264_ epitope of TMEV-OVA [5]. We therefore evaluated potential competition amongst CD8 T cell responses during acute TMEV-OVA infection of the brain. To accomplish this, we analyzed CD8 T cell responses against the vector and recombinantly-expressed antigen following seven-day intracranial (i.c.) infection with TMEV-OVA. One day prior to vaccination, mice received silencing VP2_121-130_ peptide, silencing OVA_257-264_ peptide, or E7_49-57_ peptide as a negative control. Seven days post-infection with TMEV-OVA, brain-infiltrating lymphocytes were assessed for frequency of antigen-specific CD8 T cells (Fig 2A). We determined that silencing the CD8 T cell response against the D^b^:VP2_121-130_ epitope resulted in an enhanced K^b^:OVA_257-264_-specific CD8 T cell response (Fig 2B and 2C). Interestingly, silencing the K^b^:OVA_257-264_-specific CD8 T cell response did not significantly impact the quantity of D^b^:VP2_121-130_-specific CD8 T cells. Notably, this was not the result of a change in the total number of CD8 T cells infiltrating the brain, suggesting that only a finite number of CD8 T cells were able to enter the brain (Fig 2D). These findings demonstrate that competition among vector-specific and targeted antigen-specific CD8 T cells occurs during acute CNS infection with TMEV-OVA.

### Picornavirus vaccine efficacy against B16-OVA melanoma is unaffected by the virus vector-specific CD8 T cell response

**Fig 3:**
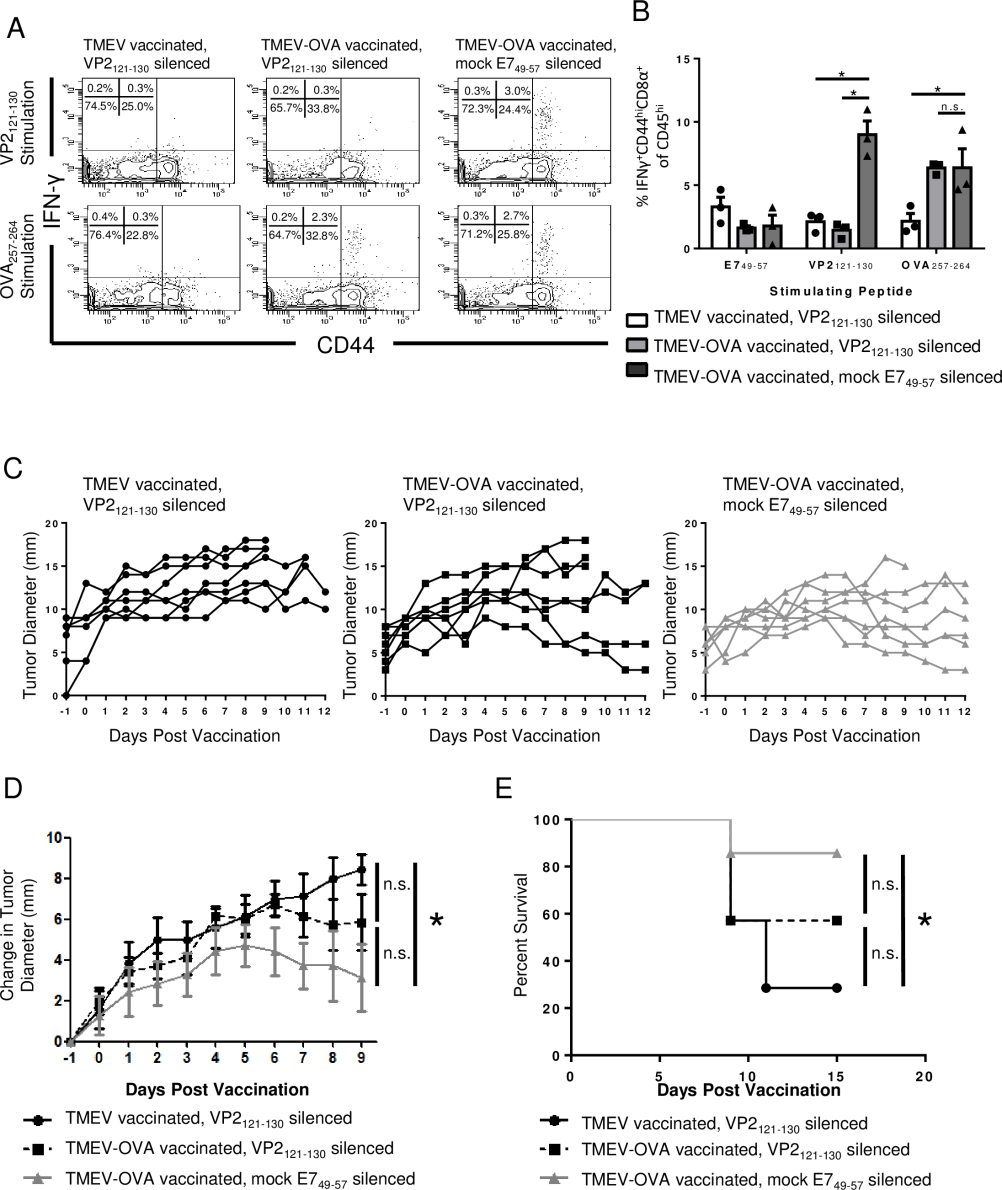
Silencing the virus vector-specific CD8 T cell response is not necessary for the function of picornavirus vaccine against B16-OVA melanoma. C57BL/6 animals were implanted with B16-OVA tumor cells in the left leg. Six days following tumor implantation, when tumors were palpable, mice were treated with vector silencing VP2_121-130_ peptide or control E7_49-57_ peptide. One day following peptide treatment, mice were vaccinated i.p. with TMEV-OVA. Splenocytes (N = 3 mice per group) were harvested from B16-OVA-bearing mice seven days after vaccination. (A) Representative flow cytometry plots and (B) mean percent of IFNγ^+^CD44^hi^ cells show a decrease in the percentage of CD8 T cells responding to VP2_121-130_ stimulation in mice administered VP2_121-130_ peptide prior to vaccination. Cells were gated as CD45^hi^ and CD8α^+^. (C) Tumor diameter, (D) change in tumor diameter, and (E) Kaplan-Meier survival curve analysis demonstrate an improvement in mice bearing B16-OVA tumors following TMEV-OVA administration compared to wild-type TMEV, regardless of silencing peptide treatment (N = 7 mice per group). Addition of the silencing VP2_121-130_ peptide does not further increase these effects. Data are shown as mean±SEM. * denotes p<0.05.

Upon confirming that virus vector-specific CD8 T cell responses could be inhibited in both the CNS and periphery, we next determined the therapeutic impact of vector silencing on picornavirus vaccination against tumors. We first assessed the impact of vector silencing on vaccination against B16-OVA melanoma. Picornavirus vaccination with TMEV-OVA has previously been demonstrated to reduce tumor burden and extend survival in B16-OVA melanoma-bearing mice [2]. We addressed the extent to which picornavirus vaccination could be improved with vector silencing. Six days post tumor implantation, animals were divided into three groups with equivalent mean tumor load. Animals then received vector silencing with VP2_121-130_ peptide. Administration of E7_49-57_ peptide served as a vector silencing negative control. The following day, all animals received i.p. picornavirus vaccination. We confirmed that D^b^:VP2_121-130_-specific CD8 T cells were silenced through administration of VP2_121-130_ peptide (Fig 3A and 3B). However, vector silencing had either no effect or a possible inhibitory effect on the K^b^:OVA_257-264_-specific CD8 T cell response (Fig 3B).

We next assessed tumor diameter in B16-OVA tumor-bearing mice. We found that control E7_49-57_ peptide administration followed by TMEV-OVA infection resulted in greater control of tumor growth compared to control TMEV treatment (Fig 3C and 3D). Additionally, VP2_121-130_-specific vector silencing prior to TMEV-OVA vaccination did not significantly reduce tumor burden or improve survival (Fig 3E). These results demonstrate that vector silencing does not positively impact tumor antigen-specific immunity or efficacy of picornavirus vaccination in the B16-OVA melanoma model.

### Vector-specific CD8 T cells enhance the tumor-specific CD8 T cell response following picornavirus vaccination against GL261 gliomas

**Fig 4:**
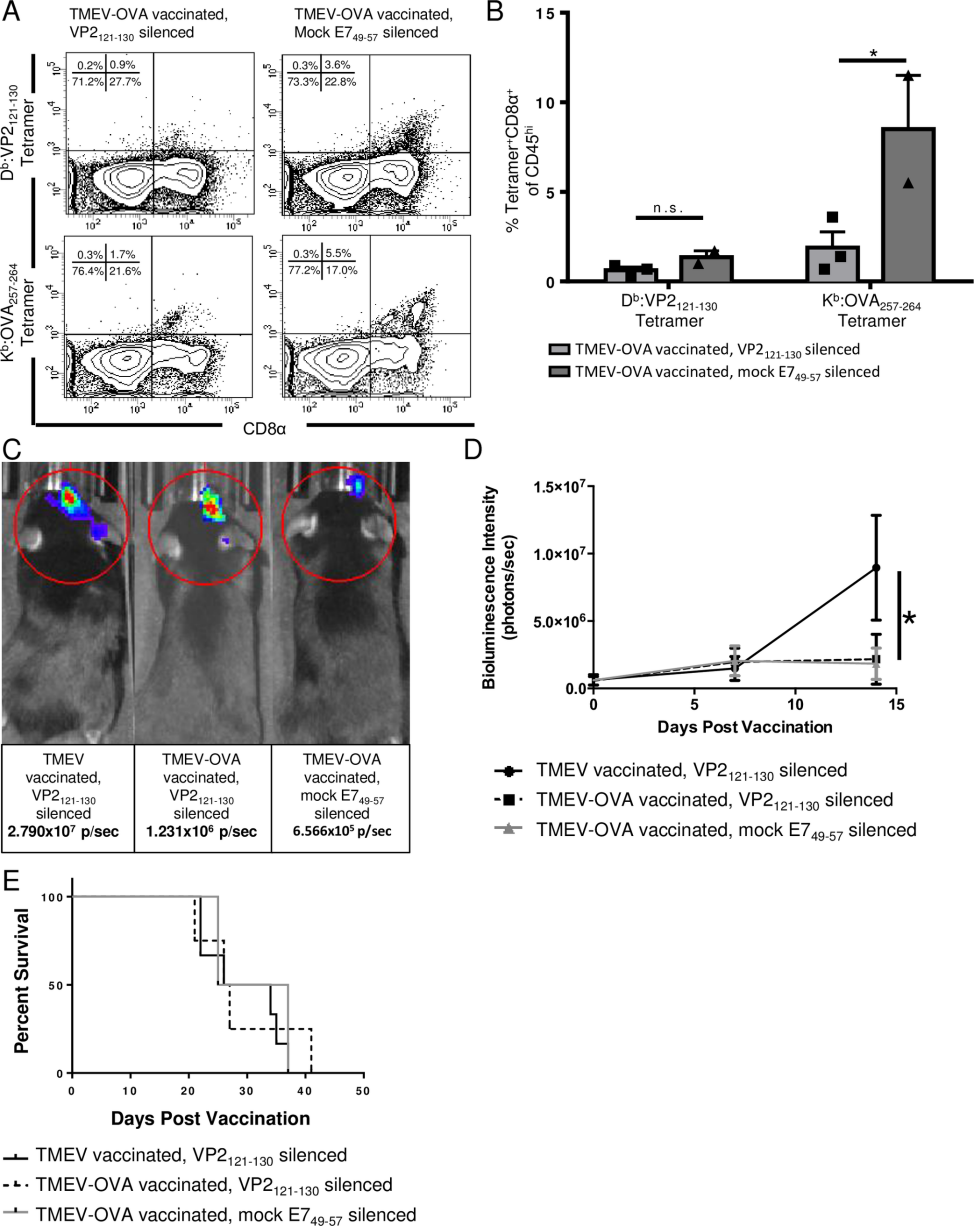
Virus vector-specific CD8 T cells enhance the tumor antigen-specific CD8 T cell response against GL261-quad cassette gliomas. GL261 quad-cassette-bearing mice (n = 7 per group) were treated with TMEV or TMEV-OVA I.C. following peptide pre-treatment with VP2_121-130_ peptide or mock E7_49-57_ peptide. Brain infiltrating lymphocytes were isolated as mice became moribund. (A) Representative flow cytometry plots and (B) percent D^b^:VP2_121-130_ and K^b^:OVA_257-264_ tetramer^+^ cells of CD8α^+^ cells isolated from the brains of GL261 quad-cassette-bearing mice demonstrate a reduction in the frequency of OVA_257-264_-specific CD8 T cells in mice receiving vector silencing to inhibit the VP2_121-130_-specific CD8 T cell response. Cells were gated as Viability Dye^lo^, CD45^hi^, and CD8α^+^. (C) Representative images and (D) quantification of tumor size by bioluminescence imaging demonstrate a significant reduction in tumor growth in mice treated with TMEV-OVA compared to control TMEV. No significant difference in control of tumor growth was observed between TMEV-OVA treated mice administered silencing VP2_121-130_ peptide or mock E7_49-57_ peptide. Data shown as mean±SEM. * denotes p<0.05.

The demonstration that vector silencing enhanced the K^b^:OVA_257-264_ epitope-specific CD8 T cell response in the brain prompted us to determine if this approach increases efficacy of picornavirus vaccination against CNS tumors. To accomplish this, we evaluated the effect of vector silencing during picornavirus vaccination against the GL261 syngeneic glioma model. GL261 glioma is a leading preclinical model of glioblastoma multiforme (GBM) for evaluating immunotherapy [23]. We implanted GL261-quad cassette glioma cells, which express OVA_257-264_, into C57BL/6 mice [5]. Two weeks post tumor implantation, tumor size was measured and mice were divided into groups with equivalent average tumor load [5,14]. Two groups then received vector silencing with VP2_121-130_ peptide. One group received mock E7_49-57_ peptide. One day following vector silencing with VP2_121-130_ peptide, mice were intracranially vaccinated with TMEV-OVA or control TMEV. Mice were imaged weekly to assess tumor size. When animals became moribund, they were euthanized and brain-infiltrating lymphocytes were isolated. The frequency of D^b^:VP2_121-130_- and K^b^:OVA_257-264_-specific CD8 T cells infiltrating the brain were quantified. We determined that TMEV-OVA vaccinated mice had low frequency of D^b^:VP2_121-130_-epitope specific CD8 T cells infiltrating the brain (Fig 4A and 4B). This finding occurred regardless of vector silencing (Fig 4B). Remarkably, we found that vector silencing with VP2_121-130_ peptide significantly reduced the targeted K^b^:OVA_257-264_-specific CD8 T cell response compared to mock E7_49-57_ peptide treatment (Fig 4B).

We next assessed the effect vector silencing had on tumor reduction in GL261-quad cassette glioma-bearing mice. We confirmed that TMEV-OVA vaccination reduced tumor burden when compared to mice treated with empty vector TMEV vaccine (Fig 4C). This is consistent with earlier reports [5,14]. Interestingly, we found no difference in tumor growth between mice receiving VP2_121-130_ peptide-induced vector silencing and mice administered sham E7_49-57_ peptide (Fig 4D). This suggests that vaccination is equally effective at controlling early GL261-quad cassette tumor growth, whether vector silencing is employed or not. Consistent with providing no changes in tumor growth, vector silencing also did not provide a marked survival advantage for mice harboring GL261-quad cassette gliomas vaccinated with TMEV-OVA, which we have previously demonstrated is, in part, mediated through immunoediting of OVA_257-264_ expression by the tumor (Fig 4E) [5]. These findings demonstrate that vector silencing has the capacity to alter, and, in the context of the GL261-quad cassette glioma, reduce, the CD8 T cell response to a targeted antigen. However, these alterations in immune responses do not translate to changes in vaccine efficacy, suggesting that, in the case of picornavirus vaccination against GL261-quad cassette gliomas, vector-specific CD8 T cell responses do not prevent an effective tumor antigen-specific response.

## Discussion

In this study, we investigated the impact of vector-specific CD8 T cells on picornavirus vaccination in both peripheral and CNS tumor models. In the process, we demonstrated that competition between CD8 T cell responses directed against different epitopes occurs during acute CNS infection. However, the results obtained from investigation of the GL261-quad cassette glioma models demonstrates that vector-specific CD8 T cells have the capacity to be additive to the immune response as a whole. This is accomplished through increasing frequency of tumor antigen-specific CD8 T cells. The differing effects of vector silencing could be attributed to factors including prior antigen exposure and microenvironment of the tumor. Future work is required to elucidate the specific contributors to differences in the outcome of vector silencing.

These findings are relevant to the use of virus vaccine platforms. Several groups using viruses engineered to express recombinant antigens have demonstrated there is a robust response to the endogenous viral antigens of other types of vectors. Specifically, recombinant vaccinia virus expressing the LCMV-derived epitope, NP_118-126_, elicited a 20- to 30-fold higher CD8 T cell response to the virus vector than the targeted antigen [7]. Additionally, vesicular stomatitis virus (VSV) expressing tumor antigens also result in VSV-specific CD8 T cell responses [4]. We have demonstrated similar findings following TMEV-OVA infection, in which we have observed the presence of CD8 T cells are directed against the encoded OVA_257-264_ antigen and the virus vector, VP2_121-130_ [3,5]. These findings are significant, as we have demonstrated the virus vector-specific CD8 T cell response may have differing effects on the antitumor immunity depending on a variety of factors.

As concern was raised over the presence of virus vector-specific CD8 T cells, additional strategies to inhibit this response were developed. Another method to silence the CD8 T cell response against the immunodominant VP2_121-130_ epitope of TMEV has been implemented through targeted mutation of the TMEV genome [24]. Bell *et al*. demonstrated that variants of TMEV in which mutations introduced into the immunodominant VP2_121-130_ epitope resulted in reduced D^b^:VP2_121-130_ epitope-specific CD8 T cell responses [24]. This was compensated by a boosted CD8 T cell response directed against the recombinantly engineered K^d^-restricted antigen, HER2/neu peptide p66 [24]. Therefore, inhibiting the virus vector-specific CD8 T cell response by mutation of immunodominant vector epitopes may have a different effect on picornavirus vaccination than vector silencing. Additionally, silencing the immunodominant epitope of a virus vector through mutation may result in a compensatory increase in subdominant epitopes of the vector. These subdominant epitopes could, in turn, enhance anti-tumor immunity. Nevertheless, based on our findings in this study, strategies aimed at reducing immunodominant epitopes of virus vectors may not be essential for therapeutic utility of virus vaccines, though this is dependent on the context of vaccination.

Picornaviruses developed as cancer treatments have demonstrated great promise as oncolytics. Ochiai *et al*. has demonstrated that poliovirus, which is highly homologous to TMEV, is an effective oncolytic therapy [25,26,27]. Administration of recombinant poliovirus to athymic rats bearing an aggressive human GBM xenograft showed efficacy compared to control treatment [26]. These findings promote the use of picornavirus therapy for GBM. However, we contend that insertion of tumor-specific antigens, such as epidermal growth factor receptor variant III (EGFRvIII) into the leader sequence of the poliovirus genome could provide additional therapeutic benefit [28,29,30]. In addition to the oncolytic properties of polio, GBM-specific CD8 T cell responses could be generated. Therefore, picornavirus vaccine strategies should be considered an attractive option for cancer therapeutics, as picornaviruses engineered to express tumor antigens have oncolytic properties and enhance antitumor immunity, which improves the tumor antigen-specific CD8 T cell response.

## Materials and Methods

### B16-OVA melanoma and GL261-quad cassette glioma cell culture and implantation

The B16-OVA melanoma and GL261-quad cassette glioma cell lines were cultured as previously described [5,14]. The B16-OVA melanoma line is engineered to express the K^b^-restricted model antigen, SIINFEKL (OVA_257-264_). The GL261-quad cassette line is engineered to express the model antigens OVA_257-264_, OVA_323-339_, human GP100_25-33_, and alloantigen I-E^a^_52-68_. The GL261-quad cassette cell line expresses luciferase to assess tumor burden using bioluminescence imaging. 5.0x10^5^ B16-OVA melanoma cells were implanted into the left hind leg of C57BL/6 mice (The Jackson Laboratory, #000664) subcutaneously in a total volume of 50μL. 6.0x10^4^ GL261-quad cassette cells were implanted by stereotactic injection into the right striatum of C57BL/6 mice (The Jackson Laboratory, #000664) as previously described [5]. Animals were anesthetized with 20mg/kg ketamine and 5mg/kg xylazine to minimize suffering for the duration of the procedure. Total injection volume was 1μL at a rate of 0.2μL per minute. Coordinates for injection site were 1mm lateral, 2mm anterior of bregma. Cells were injected a depth of 3mm from the surface of the cortex. All animal experiments were approved by and performed in accordance to the Mayo Clinic Institutional Animal Care and Use Committee.

### Administration of peptide

100.0μL of 1.0mg/mL E7_49-57_ (RAHYNIVTF) or VP2_121-130_, (FHAGSLLVFM) peptide (GenScript Biotechnology, Piscataway, NJ) were administered i.v. one day prior to vaccination with TMEV or TMEV-OVA. B16-OVA melanoma-bearing mice were treated with peptide 6 days post tumor implantation, while GL261-quad cassette-bearing mice were treated with peptide 14 days following tumor implantation. Mice assessed during acute TMEV-OVA infection received i.v. peptide one day prior to viral infection.

### Vaccination with picornavirus

The recombinant TMEV XhoI-OVA8 (TMEV-OVA) picornavirus was generated as previously described [3]. Briefly, mice receiving i.p. vaccination were administered a single dose of 2.0x10^7^ plaque forming units (PFU) wild-type TMEV or TMEV-OVA one day following peptide administration. Mice receiving i.c. vaccination were administered a single dose of 2.0x10^6^ PFU TMEV or TMEV-OVA following anesthesia with 1–2% isoflurane. GL261-quad cassette-bearing mice were vaccinated in the opposite hemisphere of the brain from where GL261-quad cassette tumor cells were implanted.

### B16-OVA tumor size and survival analysis

B16-OVA tumor size was assessed via caliper measurement (Fisher Scientific, #12-125-1). Tumor diameter was measured daily as tumors became palpable. Mice were euthanized by CO_2_ inhalation when tumors reached a diameter of 19mm, in accordance with Mayo Institutional Animal Care and Use Committee standards [3].

### Bioluminescence imaging and analysis

GL261-quad cassette tumor burden was assessed using bioluminescence imaging. Prior to imaging, animals were administered 150mg/kg D-luciferin i.p. (Gold Biotechnology, #LUCNA-250). Bioluminescence imaging was performed using an IVIS Spectrum system (Xenogen Corp., Alameda, CA, USA) running Living Image software as previously described [5,14]. Animals were anesthetized with 1–2% isoflurane during imaging. Bioluminescence signal intensity (photons/second) was quantified in a circular region of interest surrounding the head. Following treatment, mice were monitored daily and euthanized by CO_2_ inhalation when they became moribund. Symptoms included weight loss, hunched posture, and difficulty moving. All animal work was completed following the Mayo Institutional Animal Care and Use Committee guidelines.

### Intracellular cytokine staining and flow cytometry

Interferon-γ (IFNγ) stimulation was assessed by intracellular cytokine staining. 2x10^6^ splenocytes were isolated and stimulated with 10ng/mL IL-2 and 1μg/mL peptide (VP2_121-130_, OVA_257-264_, or E7_49-57_) and Golgi Plug at a concentration of 1μL/1mL media (BD Biosciences, #555028). Cells were incubated for 5hr at 37°C and 5% CO_2_, then stained for surface markers, followed by permeablization and intracellular staining for IFNγ (BD Biosciences, #555028). For CNS-infiltrating immune cells, brains were homogenized and digested with collagenase type IV (Worthington Biochemical #9001-12-1) or manual dounce homogenization (Thomas Scientific, #7722–15) and centrifuged against a percoll density gradient at 7846g for 30 minutes to isolate immune cells. Peptide:MHC tetramers were constructed as previously described [31,32]. Antibodies against CD45, CD8α, and CD44 at a 1:100 dilution (BD Biosciences, #557235, #561097, #553133), as well as ghost dye to assess viability at a 1:1000 dilution (Tonbo Biosciences, #13-0871-T500) were used for staining. Samples were run on a BD LSRII flow cytometer and analyzed with FACSDiva software (BD Biosciences, San Jose, CA). Additionally, samples were digitally compensated using single-stained controls by FACSDiva software.

### Statistical analysis

All data are presented as mean ± SEM. For comparisons of two groups, significance was determined using Student’s *t* test or Mann-Whitney Rank Sum Test if the data did not follow a normal distribution. Survival was assessed using a logrank (Mantel-Cox) test. GraphPad Prism 6.0 (La Jolla, CA) was used for statistical analysis.
